# Coherent resonance in the distributed cortical network during sensory information processing

**DOI:** 10.1038/s41598-019-54577-1

**Published:** 2019-12-04

**Authors:** Alexander N. Pisarchik, Vladimir A. Maksimenko, Andrey V. Andreev, Nikita S. Frolov, Vladimir V. Makarov, Maxim O. Zhuravlev, Anastasija E. Runnova, Alexander E. Hramov

**Affiliations:** 1 Center for Biomedical Technology, Technical University of Madrid, Pozuelo de Alarcón, Madrid, Spain; 2 Neuroscience and Cognitive Technology Laboratory, Center for Technologies in Robotics and Mechatronics Component, Innopolis University, 1 Universitetskaya str., Innopolis, The Republic of Tatarstan Russia

**Keywords:** Perception, Pattern vision, Computational science

## Abstract

Neuronal brain network is a distributed computing system, whose architecture is dynamically adjusted to provide optimal performance of sensory processing. A small amount of visual information needed effortlessly be processed, activates neural activity in occipital and parietal areas. Conversely, a visual task which requires sustained attention to process a large amount of sensory information, involves a set of long-distance connections between parietal and frontal areas coordinating the activity of these distant brain regions. We demonstrate that while neural interactions result in coherence, the strongest connection is achieved through coherence resonance induced by adjusting intrinsic brain noise.

## Introduction

It is well-known known that brain dynamically adjusts the structure of its functional neuronal network to enhance the efficiency of sensory processing under increasing cognitive demand^1,2^. This mechanism is described in the framework of the global workspace theory, which implies that conscious perception requires coherent activity of multiple distributed brain regions^3,4^. In this context, perception and preliminary processing of visual information is performed by the fronto-parietal network^5,6^. The mechanisms underlying the emergence of neural connections between remote regions of the brain are still unknown and remain a widely debated problem in neuroscience. There are several well-known theories which are about to explain how neural ensembles communicate in the brain. In particular, Fries^7^ hypothesized that neuronal communication is subserved by neuronal coherence. According to his theory, activated neuron groups communicate during temporal windows when they are coherent. Furthermore, Gregoriou *et al*.^8^ suggested that the mechanism of neuronal communication is implemented through high-frequency gamma-band oscillations. The authors showed that time-shifted coupling at gamma frequencies may optimize the post-synaptic impact of spikes from one area upon the other, and, by this, improve cross-area communication. Lisman and Jensen^9^ demonstrated that along with gamma frequencies, theta frequencies also play important role in neuronal communication. They found that gamma and theta frequency oscillations occur in the same brain regions and interact with each other coordinating communication between brain regions. Finally, it was concluded that neuronal communication is simultaneously conducted at different frequency bands and requires coherence^10^. This requirement for efficient communication known as *Communication Through Coherence* (CTC) implies that a postsynaptic neural group, receiving input signals from several presynaptic groups, responds primarily to those group with which it is more coherent. In the absence of the coherence, the inputs arrive at random phases of the excitability cycle and therefore have a very low connectivity efficiency.

Although the important role of the coherence mechanism for communication between remote brain regions was highlighted^11^, the understanding of how CTC helps the brain to perform cognitive tasks still remains an enduring challenge of modern neuroscience^12^. In this respect, we suppose that the improvement of neuronal communication can be achieved through *coherence resonance* (CR) which appears in the neuronal network at a certain level of noise. CR in neuronal networks was predicted by numerical simulations of different neural models^13–15^. CR in the brain implies the existence of intrinsic brain noise caused by internal thermal fluctuations of membrane conductance mediated by random opening and closing ion channels^16,17^. We hypothesize that the brain in some way adjusts this noise according to cognitive demand in order to increase signal-to-noise ratio.

Let us consider the Hodgkin-Huxley neuronal network model, where the dynamics of each neuron is affected by intrinsic noise. Let, the values of *S*^*pow*^ and *I*^*ext*^ represent the intensity of noise and external stimulus, respectively. In the absence of noise, the behavior of the neural ensemble is only regulated by the external signal.

**Figure 1:**
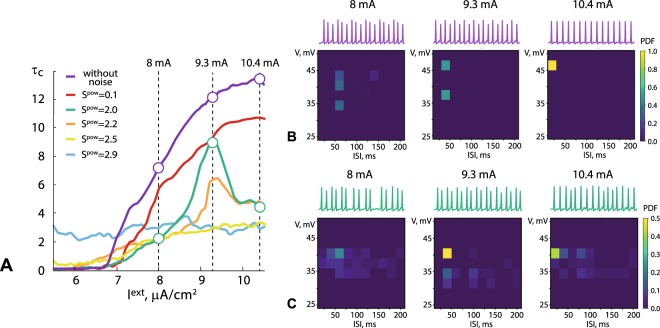
Results of the Hodgkin-Huxley network model analysis. (**A**) Correlation time ${\tau }_{c}$ versus the external signal intensity *I*^*ext*^ in the absence (purple curve) and in the presence of intrinsic noise for different values of the noise intensity *S*^*pow*^ (see legend). Macroscopic activity of the model cortical network under increasing *I*^*ext*^ in the absence (**B**) and in the presence (**C**) of intrinsic noise. The upper rows in (**B** and **C**) display typical shapes of the averaged action potential signal *V*_*arr*_, while the lower plots show PDFs of interspike intervals ISI corresponding to different spike amplitudes *V*.

Here, we are most interested in the neuronal network behavior near the excitation threshold. The coherence of the neuronal activity in the model cortical network was estimated by the correlation time ${\tau }_{c}$ (See Eq. 18). Figure 1A illustrates the dependence of ${\tau }_{c}$ on the noise intensity *S*^*pow*^ and the external signal amplitude *I*^*ext*^. In the absence of noise all the neurons are silent below the excitation threshold (${I}^{ext} < 6.8\,\mu$A/cm^2^) due to the absence of the random fluctuations enabling switching of the neuron’s activity from the silent state (fixed point) to the spiking activity (limit cycle). Increasing *I*^*ext*^ above excitation threshold results in a monotonous growth of the maximal correlation time ${\tau }_{c}$ (purple curve in Fig. 1A).

Introduction of low-intensity noise (${S}^{pow}=0.1$) reduces network’s coherence above the excitation threshold (red curve in Fig. 1A). However, network’s behavior remains qualitatively similar: coherence grows monotonically with increasing *I*^*ext*^. At the same time, strong intrinsic noise (${S}^{pow} > 2.3$) shifts excitation threshold to the lower values of *I*^*ext*^ and considerably reduces network’s coherence above the threshold (yellow and blue curves in Fig. 1A). This is due to the irregular single neuron dynamics caused by high-intensity intrinsic noise enabling frequent and random neuronal excitations^18,19^. In the particular range of the noise intensity ($1.9 > {S}^{pow} > 2.3$) the correlation time changes non-monotonically: it reaches the maximal value for a certain *I*^*ext*^ and decreases with the further growth of the external signal amplitude. In the neighborhood of the excitation threshold optimal intensity of intrinsic noise subserves the increase of network’s coherence by analogy with the mechanisms revealed in the previous numerical studies on coherent resonance in the networks of excitable units^20–24^. In this case noise maintains coherent neuronal interaction by frequent switching of single neurons to the spiking behavior. Further increase of external signal amplitude reduces the constructive effect of noise and, therefore, causes network’s coherence collapse.

The changes in network’s dynamics in the absence and the presence of intrinsic noise are illustrated in Fig. 1B,C, respectively, by the probability density function (PDF) of interspike intervals (ISI) at corresponding spike amplitudes. The network coherence can be estimated via PDF; the more pronounced PDF peak indicates higher regularity. The figures show the network behavior for three values of the external stimulus amplitude: slightly above the excitation threshold (8 *μ*A/cm^2^), at the optimal amplitude corresponding to maximum ${\tau }_{c}$ (9.3 *μ*A/cm^2^), and above the optimal amplitude (10.4 *μ*A/cm^2^). In the absence of intrinsic noise (Fig. 1B), an increase in the external stimulus intensity causes the transition from a slightly incoherent collective behavior (at ${I}^{ext}=8\,\mu$A/cm^2^) to a regular spike generation (at ${I}^{ext}=10.4\,\mu$A/cm^2^) characterized by homogeneity of both the spike amplitude and the inter-spike interval (ISI). The presence of intrinsic noise crucially changes the collective behavior in the neural network. As seen from Fig. 1C, strong heterogeneity of ISI and spike amplitudes is observed at small (${I}^{ext}=8\,\mu$A/cm^2^) and high (${I}^{ext}=10.4\,\mu$A/cm^2^) amplitudes with maximum PDF at 0.22 and 0.41, respectively, whereas the most regular collective dynamics is observed at the intermediate stimulus amplitude (${I}^{ext}=9.3\,\mu$A/cm^2^), where the highest PDF maximum (0.5) occurs.

Thus, one can see that optimal value of *I*^*ext*^ contributes to coherent network dynamics and launches neural mechanisms of stimulus perception and processing.

Increasing *I*^*ext*^ in a model neuron leads to increasing firing rate of it^25^. According to the results of neurophysiological studies, increasing image contrast also leads to increasing firing rate of neurons in visual cortex^26–30^. Thus, increasing *I*^*ext*^ in simulation can be associated with increasing of visual stimulus contrast in the neurophysiological experiment.

The above numerical results predict that intrinsic noise causes a resonant behavior of the stimulus-related brain response at the external signal amplitude slightly above the neural excitation threshold. This gives us hope for experimental discovery of this resonance effect induced by intrinsic brain noise by analyzing stimulus-related brain response to visual stimuli near the perception threshold.

**Figure 2:**
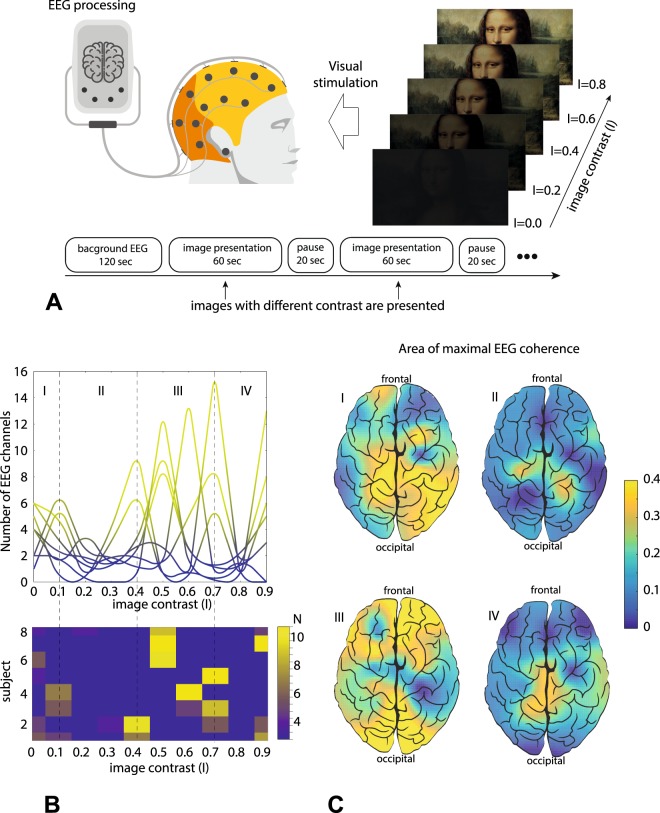
Experimental results demonstrating coherence resonance near perception thresholds. (**A**) Schematic illustration of the experimental protocol. (**B**) (Upper panel) Number of EEG channels with maximal correlation time versus image contrast for different subjects (each curve corresponds to one participant in the experiment). The most local maxima are concentrated in areas I and III corresponding to image recognition and portrait identification, respectively. (Lower panel) Distributions of the coherent channel among participants. (**C**) Brain coherence for different image contrasts: (I) $I=0.1$, (II) $I=0.4$, (III) $I=0.7$, and (IV) $I=0.8$ for one typical subject.

In accordance with the foregoing, we explore visual stimuli, a set of Mona Liza portraits, with different contrast level *I* (see Fig. 2A). Since this picture contains a lot of small details, the increasing contrast allows the observer to distinguish more and more details, so that finally the Mona Liza portrait will be completely recognized. The image recognition passes through several levels of perception, which include a low (or basic) level corresponding to perception of the Mona Liza silhouette and high perception levels related to the detailed recognition of facial features and background. Thus, the presented visual stimuli may have multiple perception thresholds.

For quantitative estimation of the coherence, we calculate the correlation time of the EEG signals. A larger correlation time of a particular EEG signal indicates a stronger coherent behavior of the corresponding neural population. Based on the numerical results, we suppose that the correlation time will exhibit a local maximum when *I* passes through a perception threshold. According to this, global network coherence can be defined as the number of EEG signals exhibiting maximal correlation time at a given contrast level. The dependence of this measure on the image contrast is shown in Fig. 2B, where different curves correspond to different subjects. One can see that global network coherence maximizes at low values of the image contrast ($I < 0.1$) and in the values of $0.3 < I < 0.7$.

In the former case (area I), the local maxima in the coherence are observed for all participants in a narrow range. It corresponds to the neural coherence induced by low-level perception of Mona Lisa silhouette. This result is confirmed by the coherent neural activity in occipital and parietal areas (see Fig. 2C). In the latter case (area III), the local maxima are distributed over a wide range of the contrast level. When the contrast is increased, an additional amount of visual information induces a sharp maximum in global network coherence. Intrinsic brain noise, individual for every subject, defines the position of the local maxima. The lower panel in Fig. 2B evidences that each subject exhibits a single local maxima in area III. Notably, the peaks in area III are much higher then in area I, i.e., the size of the neural network involved in sensory processing is maximized in area III. In Fig. 2C this case is characterized by the coherent behavior of frontal and occipital-parietal brain networks. Finally, the excitation of coherent dynamics of the distributed network is observed for certain contrast levels (area III). For other areas, where contrast values are less (area II) and higher (area IV) than the contrasts in area III, the distributed brain structure is not excited and the sensory processing engages the visual area.

**Figure 3:**
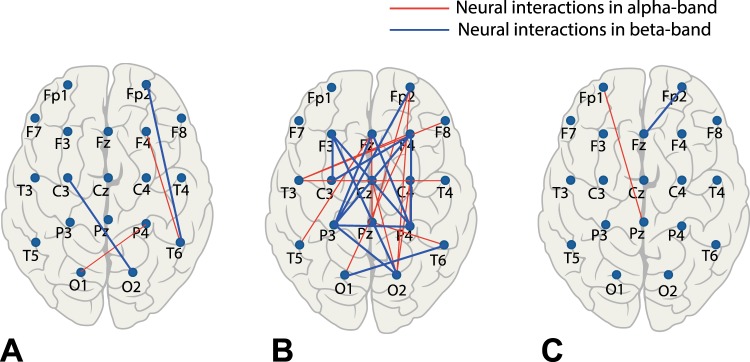
Structure of brain connectivity in alpha (8–12 Hz; red links) and beta (15–30 Hz; blue links) bands for different image contrasts: (**A**) $I=0.1$ (left column), (**B**) $I=0.4$ (middle column), and (**C**) $I=0.7$ (right column). The link strengths are estimated via wavelet bicoherence.

Having in mind that coherence is a key mechanism for neural communication, we suggest that an increase in coherence in frontal and occipito-parietal areas contributes their effective interaction in different frequency bands. To prove this hypothesis, we reconstruct the brain network from multivariate EEG signals using wavelet bicoherence. This approach allows finding a link between a pair of brain regions in terms of synchronization between corresponding EEG signals in a particular frequency band. In Fig. 3 we show typical network structures in areas II–IV by drawing the links between EEG channels with high coherence in alpha (red links) and beta (blue links) frequency bands. Having compared these areas, one can see that in both bands the maximum number of links appears in area III (middle column) associated with resonant neural response to the visual stimulus. Network structure contains multiple links connecting frontal and parietal brain regions.

It is known that brain noise affects all nervous system functions, from perception of sensory signals to generation of motor responses^17^. In sensory processing, brain noise is usually associated with variability of neural responses to identical visual stimuli and affects visual perception at different stages. Before visual information is being processed in the brain, it is affected by sensory noise occurring in sensory signals and sensory receptors. It should be noted that visual information also contains noise caused by photons arriving to photoreceptor cells in the retina at a random rate governed by a Poisson process. Then, the noise in sensory receptors is amplified and converted into electrical signals. Sensory noise can be considered as external noise. With this in mind, the effect of external noise was studied by analyzing brain response to visual stimuli with different amount of noise. This allowed to experimentally observe stochastic resonance of the brain response by changing the external noise amplitude^31^. An increase in the signal detection efficiency in the presence of external noise was experimentally observed by Simonotto *et al*.^32^. The authors demonstrated that subjects exhibited the highest perception performance for some optimal (not zero) level of the external noise intensity. The obtained results contribute to the theory of beneficial effects of noise on sensory processing^33^.

Unlike external noise, the effect of intrinsic brain noise is purely studied, because the parameters of this kind of noise are controlled by the brain itself and cannot be varied by the experimentator. At the same time, the effect of intrinsic brain noise was studied in different neural models^14^. The obtained numerical results evidence that the optimal brain noise amplitude causes the most coherent network response to the external signal. The effect of intrinsic brain noise is actively studied in cellular systems. For example, Paszek *et al*.^34^ reported that intrinsic noise resulting in heterogeneity between individual cells contributes coordination of cell population responses to perturbation. Kellogg *et al*.^35^ showed that intrinsic biochemical noise improves oscillations and entrainment of single cells. Intrinsic noise in the brain neural network is cellular noise caused by stochastic processes which take place in neurons at the biochemical and biophysical level. They include protein production and degradation, opening and closing ion channels. Since neurons perform highly nonlinear operations, small biochemical and electrochemical fluctuations may significantly alter whole-cell responses^16^. Along with cellular noise there exists synaptic noise which appears when the neurons receive an intense synaptic bombardment from thousands of synapses^36^.

According to our study, intrinsic brain noise which influences neural brain activity on *microscopic* level has a beneficial effect on a *macroscopic* level. Namely, it coordinates responses of different brain areas and makes them to cooperate for efficient processing of sensory information. The questions then are whether parameters of intrinsic brain noise are constant or evolve in time and which mechanisms underlie variation of its parameters.

First, intrinsic brain noise is supposed to be unique characteristic of a subject, which defines within subjects variability during simple cognitive task accomplishing^37^. At the same time, the recent work suggests that perceptual abilities can be improved by cognitive training accompanied by the reduction of noise correlation^38^. Thus, according to the recent result, indistinct brain noise being individual characteristic of a subject can vary as a result of cognitive training in order to optimize the efficiency of the task-related brain response. The possibility to change the noise structure gradually by the training is not surprising. It is known that cognitive training can even induce morphological changes in the brain^39^.

The more interesting question is whether or not the brain noise parameters are instantly adjusted by the brain when cognitive demand is increased. If it is true, then the reasonable question is how to control brain noise in order to help a person to process sensory information in a most efficient way, during the accomplishment of cognitive tasks. The first work on this topic was published in 2018 by Huidobro *et al*.^40^. The authors used optogenetic brain stimulation to vary the noise level in neuronal population in barrel cortex of mice. As a result, they found that noise stimulation increases the neuronal multiunit-activity response evoked by whiskers stimulation. This finding suggests that optimal non-zero intensity of noise stimulation could produce improvements in somatosensory perception. Some months later, van der Groen *et al*.^41^ applied this idea in humans. They added noise directly to the visual cortex using transcranial random noise stimulation (tRNS) while participants accomplished dot-motion discrimination task. It was shown that adding an optimal amount of noise bilaterally to the visual cortex can enhance perceptual decision-making.

Having summarized, the experimental and theoretical studies provide substantial evidence for the beneficial effect of intrinsic brain noise on the efficiency of sensory processing and cognitive ability. At the same time, the effect of noise is observed for neuronal ensembles in particular task-related areas, mostly in visual cortex. According to our study, intrinsic brain noise contributes not only for enhancing neuronal response in particular brain areas, but also provides pathways for neural communication between remote brain regions. In this context, our study is about to bridge the gap between neural noise paradigm and neural communication theories (Communication Through Coherence and Global Workspace Theory). Our results confirm other studies claiming that effective visual sensory processing in the brain requires neural communication within the frontoparietal cortical network and that the neural communication requires coherence. In addition, we suppose that the coherence can be achieved through the coherent resonance subserved by the presence of intrinsic brain noise. Taking into account that the present study is based on the small group of participants, the future studies will have to confirm this proof-of-concept exploiting a large sample of subjects and conservative statistical models.

## Methods

### Participants

Twenty healthy unpaid volunteers, 12 males and 8 females, between the ages of 20 and 43 with normal or corrected-to-normal visual acuity participated in the experiments. All of them provided informed written consent before participating. The experimental studies were performed in accordance with the Declaration of Helsinki and approved by the local research Ethics Committee of the Yuri Gagarin State Technical University of Saratov.

### Experimental procedure

In our experiments, we used the Mona Liza portrait as a visual stimulus presented to every participant (Fig. 1). The color images with different brightness values *I* were demonstrated during 60-s time intervals on the 24″ BenQ LCD monitor with a resolution of 1920 × 1080 pixels and a refresh rate of 60 Hz. The monitor was located at a distance of 70–80 cm with a visual angle of approximately 0.25 rad. To draw away the observer’s attention and provide some time for rest, there were 20-s time windows between subsequent demonstrations of the Mona Liza portraits.

All participants were instructed to focus their attention on the pictures during each presentation. The whole experiment lasted around 15 min for each participant, including 120-s recordings of the brain background activity before and after the stimuli presentations. During the experimental sessions, the pictures with different brightness *I* were randomly presented and electrical brain activity was simultaneously recorded using the amplifier BE Plus LTM, manufactured by EB Neuro S.p.a., Florence Italy (www.ebneuro.com). The monopolar registration method and classical 10–20 electrode system were used.

In the experiment, we used 10 different values of *I* from 0.1 to 1.0 with a step of 0.1. The values of $I=0$ and $I=1$ correspond, respectively, to 0% and 100% of natural pixels’ luminance of the picture.

### Connectivity

The brain connectivity was revealed from the analysis of EEG signals recorded by $M=19$ electrodes (see Table 1) placed on standard positions of the 10–20 international system (Fig. 2) using continuous wavelet transformation. From each time series ${x}_{p}(t)$ from *p*-th electrode, we calculated the wavelet energy spectrum in the frequency range $f\in [0,30]\,{\rm{Hz}}$ as follows

1 $${E}^{p}(f,t)=\sqrt{f{[{\int }_{t-4/f}^{t+4/f}{X}_{p}(t){\psi }^{\ast }(f,t)dt]}^{2}},$$

where $\psi (f,t)$ is a mother wavelet function and “*” denotes complex conjugation. As a mother wavelet function, we choose the Morlet wavelet, often used for the analysis of neurophysiological data, defined as

2 $$\psi (f,t)=\sqrt{f}{\pi }^{1/4}\,{{\rm{e}}}^{j{\omega }_{0}f(t-{t}_{0})}\,{{\rm{e}}}^{f{(t-{t}_{0})}^{2}/2},$$

where ${\omega }_{0}=2\pi$ is the wavelet parameter and *t*_0_ is the shift of the wavelet along the time axis.

**Table 1:** Parameters of the Experiment.

Parameter	Value
Time interval of background EEG recording	120 sec
Time interval of each visual stimuli presentation	60 sec
Time interval between visual stimuli presentations	20 sec
Number of presented visual stimuli	10
Total duration of the experimental session	920 sec
Location of EEG scalp electrodes	International 10–20 system
EEG recording sampling rate	250 Hz
EEG recording filtering	1–30 Hz
Considered EEG channels	*O*_1_, *O*_2_, *P*_3_, *P*_4_, *P*_*z*_, *C*_3_, *C*_4_, *C*_*z*_, *F*_3_, *F*_4_, *F*_*z*_, *T*_3_, *T*_4_, *T*_5_, *T*_6_, *F*_7_, *F*_8_, *Fp*_1_, *Fp*_2_
Considered EEG bands	*α*-waves (8–12 Hz), *β*-waves (15–30 Hz)

To estimate the degree of interaction between each two EEG channels (${x}_{p}(t)$ and ${x}_{q}(t)$) via wavelet bicoherence, we considered the corresponding complex wavelet coefficients ${W}_{p}(f,t)={a}_{p}(f,t)+i{b}_{p}(f,t)$ and ${W}_{q}(f,t)\,=$ ${a}_{q}(f,t)+i{b}_{2}(f,t)$ and calculated signal phases ${\phi }_{p}(f,t)=\arctan \frac{{b}_{p}(f,t)}{{a}_{p}(f,t)}$ and ${\phi }_{q}(f,t)=\arctan \frac{{b}_{q}(f,t)}{{a}_{q}(f,t)}$. Then, we found the relative phase difference $\Delta \phi (f,t)={\phi }_{q}(f,t)-{\phi }_{p}(f,t)$ and obtain the coefficients

3 $$\cos \,\Delta \phi (f,t)=\frac{{a}_{p}(f,t){a}_{q}(f,t)+{b}_{p}(f,t){b}_{q}(f,t)}{\sqrt{{a}_{p}^{2}(f,t)+{b}_{p}^{2}(f,t)}\sqrt{{a}_{q}^{2}(f,t)+{b}_{q}^{2}(f,t)}},$$

4 $$\sin \,\Delta \phi (f,t)=\frac{{a}_{q}(f,t){b}_{p}(f,t)-{a}_{p}(f,t){b}_{q}(f,t)}{\sqrt{{a}_{p}^{2}(f,t)+{b}_{p}^{2}(f,t)}\sqrt{{a}_{q}^{2}(f,t)+{b}_{q}^{2}(f,t)}},$$

which we averaged over a full length of each *i*-th stimulus presentation (${\tau }_{i}=60\,{\rm{s}}$). As a result, we obtained the coefficients for *i*-th stimulus:

5 $$A={\int }_{{\tau }_{i}}\,\cos \,\varDelta \phi (f,t)dt,$$

6 $$B={\int }_{{\tau }_{i}}\,\sin \,\Delta \phi (f,t)dt,$$

and calculated the degree of coherence between every pair of EEG signals during *i*-th stimulus presentation, as the amplitude of mutual wavelet spectrum:

7 $$\sigma {(f)}_{{\tau }_{i}}=\sqrt{{A}^{2}+{B}^{2}}.$$

This function takes values between 0 and 1. If $\sigma {(f)}_{{\tau }_{i}}=0$, then there is no phase coherence between the signals at frequency *f*, otherwise, the coherence takes place.

Next, we averaged the values of $\sigma {(f)}_{{\tau }_{i}}$ over *α* (8–12 Hz) and *β* (15–30 Hz) frequency bands. Finally, we calculated the coherence between every pair of EEG signals during visual perception in the *α* and *β* frequency bands as

8 $${\sigma }_{{\tau }_{i}}^{\alpha ,\beta }={\int }_{\alpha ,\beta }\,\sigma {(f)}_{{\tau }_{i}}df.$$

### Numerical model

Our numerical model based on the Hodgkin-Huxley (HH) neurons describes a simplified bottom-up process of visual stimulus processing. It is known, that a visual sensory input excites thalamic neurons, that in turn activate larger neuronal populations of visual cortex. In the numerical simulations we considered a small network of ${N}^{ext}=5$ neurons in the thalamus and a larger network of $N=50$ neurons in the visual cortex. The thalamic neurons were biased by the external current *I*^*ext*^ associated with visual stimulus contrast. Within both thalamic and cortical networks all neurons were connected to each other, while the thalamic neurons were linked unidirectionally to the cortical neurons with a 30% probability. The network dynamics was evaluated by analyzing the event-related potentials averaged over all cortical neurons.

As mentioned above, each network unit was described by the physiological Hodgkin-Huxley model

9 $$\begin{array}{rcl}{C}_{m}\frac{d{V}_{j}}{dt} & = & -{g}_{Na}^{max}{m}_{j}^{3}{h}_{j}({V}_{j}-{V}_{Na})-{g}_{K}^{max}{n}_{j}^{4}({V}_{j}-{V}_{K})\\  &  & -\,{g}_{L}^{max}({V}_{j}-{V}_{L})+{I}_{j}^{ext}+{I}_{j}^{syn},\end{array}$$

where ${C}_{m}=1\,\mu$F/cm^3^ is the capacity of cell membrane, ${I}_{j}^{ext}$ is an external bias current injected into *j*-th neuron in the network, *V*_*j*_ is the membrane potential of *j*-th neuron. The coefficients ${g}_{Na}^{max}=120\,{\rm{mS}}/{{\rm{cm}}}^{2}$, ${g}_{K}^{max}=36\,{\rm{mS}}/{{\rm{cm}}}^{2}$ and ${g}_{L}^{max}=0.3\,{\rm{mS}}/{{\rm{cm}}}^{2}$ respectively denote the maximal sodium, potassium and leakage conductance when all ion channels are open. ${V}_{Na}=50\,{\rm{mV}}$, ${V}_{K}=-\,77\,{\rm{mV}}$ and ${V}_{L}=-\,54.4\,{\rm{mV}}$ are the reversal potentials for sodium, potassium and leak channels, respectively. *m*, *n* and *h* represent the mean ratios of open gates of specific ion channels. *n*^4^ and *m*^3^*h* are the mean portions of open potassium and sodium ion channels within a membrane patch. The dynamics of the gating variables ($x=m,n,h$) is described as follows

10 $$\frac{d{x}_{j}}{dt}={\alpha }_{{x}_{j}}({V}_{j})(1-{x}_{j})-{\beta }_{{x}_{j}}({V}_{j}){x}_{j}+{\xi }_{{x}_{j}}(t),\,\,(x=m,n,h),$$

where *α*_*x*_(*V*) and *β*_*x*_(*V*) are rate functions defined as

11 $${\alpha }_{{m}_{j}}({V}_{j})=\frac{0.1(25-{V}_{j})}{{{\rm{e}}}^{(25-{V}_{j})/10}-1},\,{\alpha }_{{n}_{j}}({V}_{j})=\frac{0.01(10-{V}_{j})}{{{\rm{e}}}^{(10-{V}_{j})/10}-1},\,{\alpha }_{{h}_{j}}({V}_{j})=0.07{{\rm{e}}}^{-{V}_{j}/20},$$

12 $${\beta }_{{m}_{j}}({V}_{j})=4{{\rm{e}}}^{-{V}_{j}/18},\,{\beta }_{{h}_{j}}({V}_{j})=\frac{1}{1+{{\rm{e}}}^{(30-{V}_{j})/10}},\,{\beta }_{{n}_{j}}({V}_{j})=0.125{{\rm{e}}}^{-{V}_{j}/80},$$

and ${\xi }_{x}(t)$ is independent zero mean Gaussian white noise, whose autocorrelation functions are

13 $$\langle {\xi }_{{m}_{j}}(t){\xi }_{{m}_{j}}(t^{\prime} )\rangle =\frac{2{\alpha }_{{m}_{j}}{\beta }_{{m}_{j}}}{{N}_{Na}({\alpha }_{{m}_{j}}+{\beta }_{{m}_{j}})}\delta (t-t^{\prime} ),$$

14 $$\langle {\xi }_{{h}_{j}}(t){\xi }_{{h}_{j}}(t^{\prime} )\rangle =\frac{2{\alpha }_{{h}_{j}}{\beta }_{{h}_{j}}}{{N}_{Na}({\alpha }_{{h}_{j}}+{\beta }_{{h}_{j}})}\delta (t-t^{\prime} ),$$

15 $$\langle {\xi }_{{n}_{j}}(t){\xi }_{{n}_{j}}(t^{\prime} )\rangle =\frac{2{\alpha }_{{n}_{j}}{\beta }_{{n}_{j}}}{{N}_{K}({\alpha }_{{n}_{j}}+{\beta }_{{n}_{j}})}\delta (t-t^{\prime} ).$$

Here, ${N}_{Na}={\rho }_{Na}S$ and ${N}_{K}={\rho }_{K}S$ represent the total number of sodium and potassium channels within membrane patch (${\rho }_{Na}=60\,\mu$m^−2^ and ${\rho }_{K}=18\,\mu$m^−2^ being sodium and potassium channel densities, respectively) and *S* is the membrane patch area of each neuron. Here, $S={10}^{-{S}^{pow}}$, where *S*^*pow*^ defines the level of noise in the model HH-neuron. The larger values of *S*^*pow*^ determine higher noise intensity and vice versa.

In Eq. (9), ${I}_{j}^{syn}$ is the total synaptic current received by neuron *j*. In this work, for simplicity we consider synaptic coupling via chemical synapses only, so that the synaptic current takes the following form

16 $${I}_{j}^{syn}=\sum _{k\in neigh(j)}\,{g}_{c}\sigma (t-{t}_{0}^{k})({E}_{rev}-{V}_{j}),$$

where the function *σ*(*t*) describes temporal evolution of the synaptic conductance, *g*_*c*_ is the maximal conductance of the synaptic channel and ${t}_{0}^{k}$ is the time at which the neighboring presynaptic *k*-th neuron fires. We suppose that $\sigma (t)={{\rm{e}}}^{-t/{\tau }_{syn}}\Theta (t)$ is proportional to the Heaviside step function $\Theta (t)$ and ${\tau }_{syn}=3\,{\rm{ms}}$.

The macroscopic network dynamics was analyzed by considering the stimulus-related potential *V*_*avr*_ averaged over the cortical neurons

17 $${V}_{avr}=\mathop{\sum }\limits_{i=1}^{N}\,{V}_{i}/N.$$

Following ref. ^42^, coherence of neuronal activity was estimated by the correlation time

18 $${\tau }_{c}={\int }_{{T}_{0}}^{{T}_{max}}\,C{(\tau )}^{2}d\tau ,$$

where ${T}_{0}=200\,{\rm{ms}}$ is the transient time, ${T}_{max}=2000\,{\rm{ms}}$ is the maximal simulation time and $C(\tau )$ is the autocorrelation function defined as

19 $$C(\tau )=\frac{\langle ({V}_{avr}(t)-\langle {V}_{avr}\rangle )\,({V}_{avr}(t+\tau )-\langle {V}_{arv}\rangle )\rangle }{\langle {({V}_{avr}(t)-\langle {V}_{avr}\rangle )}^{2}\rangle },$$

where $\langle \ldots \rangle$ is the time average after transients. The larger the ${\tau }_{c}$, the better the regularity (or coherence).
